# Impact of epoetin *β* on quality of life in patients with malignant disease

**DOI:** 10.1038/sj.bjc.6600801

**Published:** 2003-04-01

**Authors:** M Boogaerts, B Coiffier, C Kainz, the Epoetin β QOL Working Group

**Affiliations:** 1 Labo Hematologie Transplant, U.Z. Gasthuisberg, Herestraat 49, B-3000 Leuven, Belgium; 2 Centre Hospitalier Lyon-Sud, Pierre-Bénite, Lyon, France; 3 Universitäts Frauenklinik Wien, Vienna, Austria

**Keywords:** erythropoietin, recombinant, quality of life, neoplasms, anaemia, factors, predisposing

## Abstract

This open-label, prospective study was conducted to compare the impact of epoetin *β* *vs* standard care on quality of life (QoL) in anaemic patients with lymphoid or solid tumour malignancies. A total of 262 anaemic patients (haemoglobin [Hb]⩽11 g dl^−1^) were randomised to a 12-week treatment with s.c. epoetin *β* (initial dose 150 IU kg^−1^ three times weekly) or standard care. Transfusions were recommended for both groups at an Hb threshold of 8.5 g dl^−1^. The primary efficacy variables were improvement in QoL as measured using the Short-Form-36 physical component summary (SF-36 PCS) score and the Functional Assessment of Cancer Therapy fatigue and anaemia subscales (FACT-F and FACT-An). A visual analogue scale (VAS) was also used as a global QoL measure. Clinical response was defined as a ⩾2 g dl^−1^ increase in Hb level without need of transfusion after the initial 4 weeks of treatment. Baseline to final visit changes in SF-36 PCS, FACT-F and VAS scores were significantly greater with epoetin *β* than with standard care (*P*<0.05); changes in FACT-An subscale score tended to be greater with epoetin *β* (*P*=0.076). Epoetin *β* significantly increased Hb concentrations relative to standard care (responders: 47% *vs* 13%; *P*<0.001). Levels of endogenous erythropoietin <50 mIU ml^−1^ were significantly predictive of response (OR 2.496, 95% CI: 1.21–5.13). Epoetin *β* therapy significantly improves QoL compared with standard care in anaemic patients with solid tumours and lymphoid malignancies.

Anaemia is a common problem in malignancy. Studies indicate that approximately 20–50% of cancer patients undergoing chemotherapy will require blood transfusions for anaemia (Skillings *et al*, 1993; Manegold, 1998). This percentage may be markedly increased with dose intensification and multiple cycles of chemotherapy (Groopman and Itri, 1999) such that, in those treated with platinum-based regimens, the frequency of transfusion requirements can reach 100% (Kaye *et al*, 1992; Nowrousian, 1998). The clinical manifestations of anaemia, such as fatigue, vertigo, dyspnoea, loss of appetite, inability to concentrate and cardiovascular problems, impair patients' functional status and sense of well-being. Typically, energy levels and exercise tolerance are considerably reduced, everyday chores become a burden and social activities are curtailed (Curt *et al*, 2000). Moreover, unrelieved anaemia is a negative prognostic factor for outcome of cancer treatment, such that anaemic cancer patients have a higher rate of relapse and mortality at the same stage of disease compared with patients who are not anaemic (Moullet *et al*, 1998; Grogan *et al*, 1999; Caro *et al*, 2001). Considerable impetus therefore exists to provide effective management of anaemia in patients with cancer.

Until recently, blood transfusion therapy represented the sole treatment option for relief of cancer- or chemotherapy-related anaemia. However, transfusion is only effective in the short term, has associated risks (e.g. alloimmunisation and risk of transmission of infection) and is subject to limitations in blood supply. Thus, blood transfusions should be reserved for cases of life-threatening anaemia. In contrast, treatment with recombinant human erythropoietin (epoetin) leads to a long-lasting increase in the number of erythroid progenitor cells and red blood cell count, and is effective and safe for the alleviation of anaemia and transfusion requirements in patients with cancer (Cazzola *et al*, 1995; Rose *et al*, 1995; Dammacco *et al*, 1998; ten Bokkel Huinink *et al*, 1998). Epoetin also improves quality of life (QoL) in anaemic cancer patients; however, only a few randomised studies have shown this (Dammacco *et al*, 2001; Littlewood *et al*, 2001; Österborg *et al*, 2002), with most data coming from large, nonrandomised, community-based studies (Glaspy *et al*, 1997; Demetri *et al*, 1998; Gabrilove *et al*, 2001). Thus, there is a need for additional well-designed, randomised trials to evaluate the specific QoL effects of epoetin in the setting of cancer-related anaemia. The present study was therefore undertaken to assess the impact of epoetin *β* on QoL compared with standard care in anaemic patients with lymphoid or solid tumour malignancies.

## MATERIALS AND METHODS

### Study design

This was an open-label, randomised, parallel-group, multicentre, multinational clinical trial, conducted between October 1996 and September 1998 in patients with chronic anaemia associated with malignancy. The study was performed in eight countries within several centres in each country (Austria, *n*=5; Belgium, *n*=4; France, *n*=7; Germany, *n*=1; Italy, *n*=5; South Africa, *n*=11; Sweden, *n*=2; and the UK, *n*=2), and consisted of a run-in period of up to 14 days followed by a 12-week treatment period. Clinic visits were scheduled every 3 or 4 weeks during the treatment period, according to chemotherapy regimen, and every 4 weeks for patients off chemotherapy.

Patients were randomised (1 : 1, stratified according to centre) to receive either epoetin *β* or standard care (control) with transfusion support. Epoetin *β* (NeoRecormon®, F Hoffmann-La Roche Ltd, Basel, Switzerland) was administered s.c., by the patients themselves, relatives or health-care personnel, commencing at a dose of 150 IU kg^−1^ three times weekly. The dose of epoetin *β* was increased to 300 IU kg^−1^ for those patients in whom haemoglobin (Hb) levels increased by <0.5 g dl^−1^ after 3–4 weeks or <1 g dl^−1^ after 6–8 weeks. The dose was reduced by 50% if the Hb level increased by >2 g dl^−1^ per month, while treatment was interrupted if Hb levels increased to >14 g dl^−1^ (treatment was recommenced at half the previous dose once the Hb level had declined to <12 g dl^−1^). Oral iron supplementation (200–300 mg elemental iron day^−1^) was recommended for those patients in whom transferrin saturation was <15%. Clinical outcomes were collected at each postbaseline visit during the 12-week treatment period. QoL was assessed at baseline, after 3–4 and 6–8 weeks' treatment and at study end. An Hb level of 8.5 g dl^−1^ was used as a guide to initiate transfusion throughout all the centres involved in the study.

The study was performed in accordance with the latest revisions to the Declaration of Helsinki and Good Clinical Practice, the study protocol having been approved by a local independent Ethics Committee of each centre.

### Patients

Adult outpatients with anaemia (Hb ⩽11 g dl^−1^) associated with multiple myeloma, non-Hodgkin's lymphoma or chronic lymphocytic leukaemia and any solid tumour treated with myelosuppressive chemotherapy, with at least three cycles remaining, were eligible for study inclusion. In addition, patients were required to have a WHO performance status of ⩽2 and a life expectancy of >6 months. Patients with anaemia arising for other reasons (iron or vitamin B_12_ deficiency, acute bleeding, haemolytic anaemia), refractory hypertension, severe renal insufficiency (serum creatinine of >2.5 mg dl^−1^ (>220 *μ*mol l^−1^)), epilepsy or acute infection were excluded, as were pregnant or lactating women and women of childbearing age who were practising unreliable contraception. Any patient scheduled to undergo bone marrow or peripheral stem cell transplantation during the study period or 4 weeks prior to the study was also excluded. Written informed consent was obtained from all patients prior to the conduct of study-related procedures.

## EFFICACY CRITERIA

### Primary variables

The primary efficacy parameters were the changes, from baseline to final visit (week 12), in the Short-Form 36 physical component summary (SF-36 PCS; Ware *et al*, 1995) score, and the anaemia and fatigue subscale scores (FACT-An and FACT-F, respectively) (Cella, 1997; Yellen *et al*, 1997) of the Functional Assessment of Cancer Therapy (FACT) anaemia and fatigue questionnaire. All QoL assessments were performed immediately prior to clinic visits so that the patients could not be influenced by references to Hb levels. The translations used for the SF-36 and FACT–F questionnaires were fully validated.

The SF-36 health survey is a 36-item self-rated instrument assessing eight domains of health functioning: physical functioning, role functioning related to physical status, bodily pain, general health, vitality, social functioning, role functioning regarding emotional status and mental health. The eight dimensions can be combined into a physical component summary (PCS) and a mental component summary (MCS) score, both obtained by adding weighted combinations of the eight subscale scores. Scores are then converted to standardised T scores that have a mean of 50 and an s.d. of 10 (Ware *et al*, 1994). The FACT-An was composed of seven items covering anaemia-related symptoms common to cancer patients: walking difficulties, dizziness, headache, shortness of breath, chest pain, lack of interest in sex and lack of motivation for normal activities. The FACT-F consisted of 13 items related to fatigue: fatigue, weakness, listlessness, tiredness (four items), energy, ability to perform daily activities, limitation of social activities (three items) and need for sleep during the day.

### Secondary variables

Change from baseline to final visit in a visual analogue scale (VAS) was utilised as a global measure of QoL. The linear VAS comprised a 13.5 cm dual polarity straight line with the anchors ‘worst imaginable health state’ and ‘best imaginable health state’ at either end. Patients were asked to assess their global QoL state over the previous week and mark the line accordingly. The QoL assessments were completed by the patient without assistance from the physician. The FACT-An global score was also assessed as a secondary efficacy parameter.

The clinical efficacy criterion was clinical response, defined as an increase in Hb concentration of ⩾2 g dl^−1^ during the treatment phase without transfusion requirement after the initial four treatment weeks. The haematopoietic response (increase in Hb of ⩾2 g dl^−1^ or an increase to ⩾12 g dl^−1^) was also assessed. Changes in Hb level between baseline and final visits (independent variable) and corresponding changes in the primary QoL parameters (SF-36 PCS, FACT-F, FACT-An and VAS; dependent variables) were determined. Other variables assessed included haematocrit (Hct), transfusion requirements, WHO performance status, iron status, Hb nadirs and endogenous erythropoietin level including the observed/predicted (O/P) log ratio (Beguin *et al*, 1992). All blood samples were taken prior to administration of chemotherapy (if applicable) and study drug, as well as before blood transfusions.

### Safety

Clinical adverse events were recorded throughout the study and evaluated by the investigator in terms of causal relation to study medication. Laboratory safety parameters included serum creatinine and platelet, leukocyte, neutrophil and lymphocyte counts. Resting systolic and diastolic blood pressures were recorded at each visit. The number of hospitalisations in each group was recorded, including whether or not they were related to anaemia.

### Statistics

Sample size calculations were based on the expected change in SF-36 PCS score (Ware *et al*, 1995; Yellen and Cella, 1995). Thus, to detect a between-group difference in SF-36 PCS score of at least four points, assuming an s.d. of 10 using a two-sided test with a statistical power of 80% and *α*=2.5%, at least 121 patients/group were required to complete the study and be evaluable for efficacy. To allow for dropouts, approximately 310 patients were to be enrolled. However, this target population could not be achieved despite prolongation of the recruitment period and expansion of the number of participating centres.

A psychometric evaluation was performed to evaluate how well the QoL scale items satisfied the assumptions underlying the Likert method for summated rating. The internal consistency reliability of each scale score was estimated using Cronbach's *α*. Cronbach's *α*, which ranges from 0 to 1, where ‘1’ equals perfect reliability, is based on the average inter-item correlation and the number of items. Minimum values equal to or greater than 0.70 have been recommended for group level comparisons (Nunnaly, 1978).

For QoL assessments only patients for whom values were available at baseline and at least one follow-up visit were included in the analysis. The data are presented in its raw form and using the last observation carried forward (LOCF) approach, for patients with missing values at the final visit. For the percentage of clinical responders, Kaplan–Meier estimates and corresponding confidence intervals (CIs) for time to treatment response were determined, and curves were compared using the log-rank test. The O/P log serum erythropoietin ratio was derived from reference regression at the particular Hct or Hb level, and was calculated for responders and nonresponders to epoetin *β*. The relation between endogenous erythropoietin level and response to treatment was explored using the odds ratio (OR) and relative risks (RR) (Cazzola and Beguin, 1992).

Appropriate parametric and nonparametric tests were used to analyse between-group differences for continuous and categorical variables, respectively. All tests were two sided and *P*<0.05 was considered significant. Assessment of statistical significance was not adjusted for multiple comparisons.

## RESULTS

### Patient demographics

The intention-to-treat population comprised 262 patients aged 24–85 (median 62) years who were enrolled and randomised to receive either epoetin *β* (*n*=133) or standard care (*n*=129). The baseline demographic/clinical and QoL characteristics of the two treatment groups are summarised in Table 1

**Table 1:** Demographic and baseline characteristics of the intention-to-treat population

	**Epoetin *β* (*n*=133)**	**Control (*n*=129)**
Sex, *n* (%)		
Male	46 (35)	52 (40)
Female	87 (65)	77 (60)
		
Age (year)		
Median (range)	62 (24–85)	62 (24–85)
		
WHO Performance Status, *N* (%)		
0	36 (27)	41 (32)
1	69 (52)	64 (50)
2	27 (20)	24 (19)
		
Time from diagnosis of anaemia of malignancy (months)		
Median (range)	2.1 (0–99)	2.7 (0–102)
		
Underlying cancer type, *N* (%)		
Lymphoid tumour	74 (56)	71 (55)
Multiple myeloma	23 (17)	22 (17)
Non-Hodgkin's lymphoma	33 (25)	28 (22)
Chronic lymphocytic leukaemia	14 (11)	20 (16)
Other	4 (3)	1 (<1)
		
Solid tumour	59 (44)	58 (45)
Ovarian	26 (20)	19 (15)
Bone, connective tissue, skin and breast	9 (7)	12 (9)
Digestive organs, peritoneum	5 (4)	7 (5)
Respiratory and intrathoracic organs	7 (5)	6 (5)
Other	12 (9)	14 (11)
Patients with chemotherapy before study, *N* (%)	90 (68)	103 (80)*
Chemotherapy		
Alkylating agents	40 (30)	40 (31)
Antimetabolites	25 (19)	25 (19)
Plant alkaloids/other natural products	38 (29)	53 (41)
Cytotoxic antibiotics/related substances	25 (19)	27 (21)
Other antineoplastic agents	42 (32)	42 (33)
Other	3 (2)	2 (2)
Median haemoglobin level, g dl^−1^ (range)	9.0 (5–13)	9.2 (5–12)
Median serum erythropoietin level, mIU ml^−1^ (range)	54 (7–1650)	58 (5–4300)
Median serum iron level, *μ*g dl^−1^ (range)	63.7 (6–472)	78.8 (4–510)
Median transferrin saturation, % (range)	20.6 (1–97)	29.0 (2–100)

* *P*=0.025 *vs* epoetin *β* group

and Table 2

**Table 2:** Baseline quality-of-life characteristics of the intention-to-treat population

	**Epoetin *β* (*n*=133)**	**Control (*n*=129)**
Baseline quality-of-life score		
*SF-36 PCS*		
Mean (SD)	35 (8.4)	38 (9.5)
Median	35	38
Range	17–60	15–60
		
*FACT-F*		
Mean (SD)	27 (12)	31 (11)*
Median	28	33
Range	1–49	2–51
		
*FACT-An*		
Mean (SD)	20 (3.8)	21 (4.4)
Median	21	22
Range	6–27	2–28
		
*VAS*		
Mean (SD)	56 (17)	62 (17)
Median	53	60
Range	11–96	18–96

SF-36 PCS=Short-Form 36, physical component summary; FACT-F=Functional Assessment of Cancer Therapy-Fatigue; FACT-An=Functional Assessment of Cancer Therapy-Anaemia; VAS=Visual analogue scale.

* *P*=0.02 *vs* epoetin *β* group.

, respectively. Overall, there were no significant between-group differences in baseline demographics and clinical characteristics (Table 1), except for a significantly higher proportion of patients in the control group having received prior chemotherapy (80 *vs* 68%; *P*=0.025).

With respect to QoL measures, baseline scores on the FACT-An subscale were comparable between treatment groups, although those randomised to epoetin *β* therapy had lower FACT-F subscale score relative to the control group (*P*=0.02) (Table 2). Some 51 patients (19%) were withdrawn during the study (epoetin *β*, *n*=30 (23%); control, *n*=21 (16%)), 20 of them for adverse events (epoetin *β*, *n*=15 (11%); control, *n*=5 (4%)). Other reasons for withdrawal, including death, loss to follow-up, withdrawal of consent and protocol violation, were similarly distributed across the two treatment groups. The average dose of epoetin *β* over the study period was 174 IU kg^−1^ per administration.

### Efficacy

#### Quality of life

The primary efficacy population, which included patients assessable for the SF–36 PCS, FACT-F and FACT-An scores, comprised 213 patients (epoetin *β*, *n*=104; control, *n*=109).

Reliabilities assessed for SF-36 subscales varied from 0.83 to 0.90 for the pooled patient population. An exception was the General Health subscale, which exhibited Cronbach's *α* coefficient of 0.75. The reliability was sufficient for group comparisons of the SF-36 PCS. The FACT-F subscale showed high internal consistency, with reliabilities over 0.9 for every single language, and 0.93 for the pooled patient population. These and other psychometric properties, such as predictive and construct validity, were consistent with those described for the original instruments (Cella, 1997; Yellen *et al*, 1997), as reflected in the reliability of the FACT–An global score, which reached 0.92 over the total patient population assessable at baseline. In contrast, the FACT-An 7-item subscale had a Cronbach's *α* of 0.68 on the pooled patient population; however, this is consistent with the findings of the validation study (0.59 and 0.70 on initial and retest administration, respectively) (Cella, 1997).

**Figure 1:**
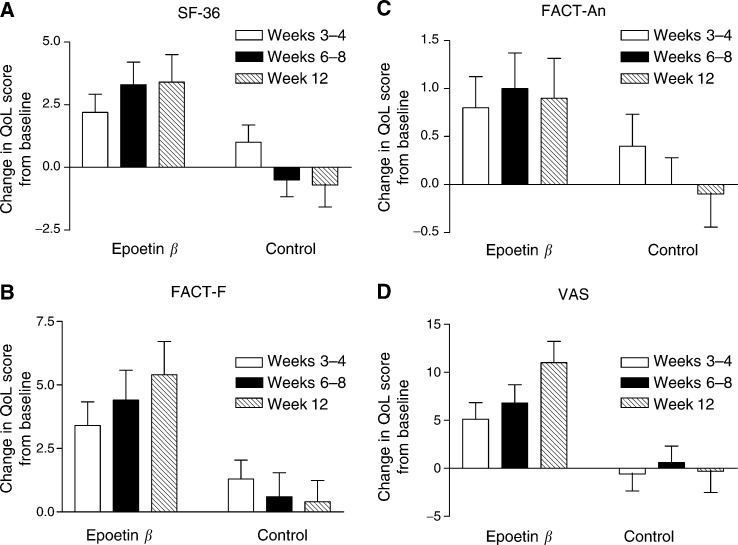
Change in quality of life (QoL) score from baseline at weeks 3–4, 6–8 and 12 during epoetin *β* therapy or blood transfusion as assessed by the SF-36 PCS, FACT-F, FACT-An and VAS instruments. Data are presented as mean (s.d.) for the patient population without last observation carried forward.

Quality of life scores for the SF-36 PCS, FACT-F and FACT-An subscales remained stable over time in the control group but significantly improved in the epoetin *β* group. Using LOCF data (*n*=104 epoetin *β*, *n*=109 control), the median changes (baseline to final visit) for the SF-36 PCS (+3.1 points) and FACT-F (+3.0 points) scores were significantly different from those in the control group (*P*<0.05), and a trend towards significance was apparent with respect to the change in the FACT-An subscale score (+1.0 points) (*P*=0.076). When the data without LOCF were used, median point increases for SF-36 PCS (+3.3 points, *n*=77, *P*=0.01) and FACT-F (+4.0 points, *n*=90, *P*=0.001) were slightly higher with the FACT-An increase remaining the same (+1.0 point, *n*=89, *P*=0.068). VAS scores remained stable over time in the control group but significantly improved in the epoetin *β* group; in the epoetin *β* group, median changes from baseline to final visit were significantly different from those in the control group (+10.0 *vs* +1.0 points, *P*=0.004 for data with LOCF (*n*=111 and *n*=112, respectively) and +10.0 *vs* +3.0 points, *P*=0.001 for data without LOCF (*n*=89 and *n*=98, respectively)). The mean change in QoL scores from baseline in the epoetin *β* and control groups for the without LOCF population are shown in Figure 1

. Similar significant results were reported for the global FACT-An score (data not shown). In the epoetin group, the rate of data points available at study end when using the raw data was 74% for the SF-36 PCS, 87% for FACT-F, 86% for FACT-An and 80% for the VAS score.

Patients with lymphoproliferative malignancies derived at least as much QoL benefit from epoetin *β* therapy as patients with solid tumours; likewise, patients previously exposed to chemotherapy showed similar QoL benefit with epoetin *β* as chemotherapy-naïve patients (data not shown). However, patients who responded to epoetin *β* therapy (i.e. achieved the target Hb response) experienced a greater improvement in QoL from baseline to final visit than patients who were nonresponders (i.e. did not achieve the target Hb response). Patients who responded to epoetin *β* therapy had a mean increase of 3.7 points in their SF-36 score, 7.2 points in their FACT-F score and 1.2 points in their FACT-An subscale scores; the corresponding improvements in the nonresponder group were 3.1, 3.4 and 0.5 points, respectively. Changes in SF-36 PCS and FACT-F scores were mediated through changes in Hb level (*P*<0.01) as shown by a path analysis where epoetin *β* treatment, QoL increase and Hb increase were used as dependent variables in turn.

#### Clinical outcomes

A significantly higher proportion of patients in the epoetin *β* group (47%) than in the control group (13%) achieved an increase in Hb of ⩾2 g dl^−1^ (*P*<0.001) (Table 3

**Table 3:** Clinical response rates (increase in haemoglobin concentration of ⩾2 g dl^−1^ and/or increase to 12 g dl^−1^ without blood transfusion) and change in haemoglobin values from baseline

	**Epoetin *β* (*n*=133)**	**Control (*n*=129)**	***P*-value**
Responders (%)			
Increase of ⩾2 g dl^−1^	63 (47)	17 (13)	<0.001
Increase of ⩾2 g dl^−1^ or increase to ⩾12 g dl^−1^	65 (49)	19 (15)	
			
Median (range) haemoglobin change from baseline (g dl^−1^)			
All patients	2.1 (−3 to 8)	0.9 (−3 to 6)	<0.001
Patients with solid tumours	2.1 (−1 to 8)	0.9 (−3 to 4)	—
Patients with lymphoid tumours	1.9 (−3 to 8)	0.9 (−3 to 6)	—
No *P*-values are provided with respect to subgroups analyses.			

). Of the 63 patients in the epoetin *β* group who responded, 44 (70%) reached the target increase in Hb level during weeks 4–8. Defining the response as an increase in Hb of ⩾2 g dl^−1^ or an increase to 12 g dl^−1^ (haematopoietic response) had only marginal effects on the number of patients responding to therapy (49 and 15% for epoetin *β* and control, respectively, *P*<0.001). A Kaplan–Meier estimate of the time to response (⩾2 g dl^−1^ only) is presented in Figure 2

**Figure 2:**
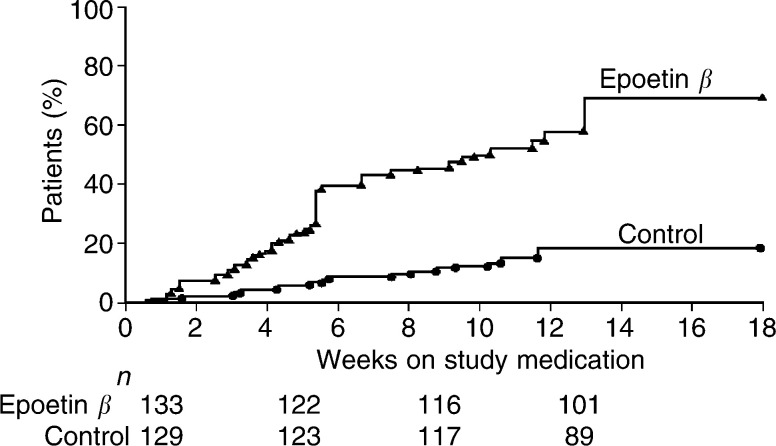
Percentage of patients showing a clinical response to therapy defined as an increase in haemoglobin ⩾2 g dl^−1^ without the need for transfusion after the initial 4 weeks of treatment.

.

**Figure 3:**
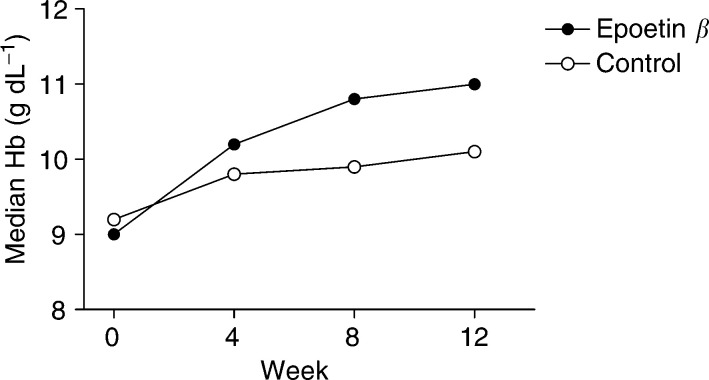
Change in median haemoglobin levels in response to epoetin *β* (*n*=133) or standard care (*n*=129) during the study period.

**Figure 4:**
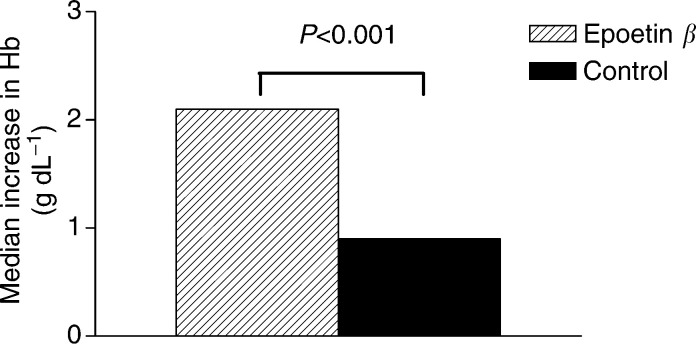
Median increase in haemoglobin levels in response to epoetin *β* (*n*=133) or standard care (*n*=129) at the end of the study period.

The median change in Hb over the study period for both groups can be seen in Figure 3

, with the overall increase shown in Figure 4

(*P*<0.001). Subgroup analyses, based on tumour and chemotherapy status, also consistently favoured epoetin *β* over control therapy (Table 4

**Table 4:** Median increase in haemoglobin level stratified by tumour type and chemotherapy status

	**Epoetin β (*n*=133)**	**Control (*n*=129)**		
	**Hb increase (g dl^−1^)**	***n***	**Hb increase (g dl^−1^)**	***n***
All patients	2.1	112	0.9	112
Solid tumour	2.1	45	0.9	51
Nonsolid tumour	1.9	67	0.9	61
With chemotherapy	2.1	74	1.0	88
Without chemotherapy	2.0	38	0.2	24

).

Threshold levels of 50 mIU ml^−1^ for serum endogenous erythropoietin and 0.9 for the O/P ratio were used in the analysis of baseline serum endogenous erythropoietin levels, as defined previously by Cazzola *et al* (1996). Endogenous erythropoietin levels of <50 mIU ml^−1^ were significantly predictive of response (OR 2.496, 95% CI 1.21–5.13). The baseline erythropoietin O/P ratio was only predictive of response for patients with nonsolid tumours. In this patient subset, 52% of patients with a baseline O/P ratio of <0.9 achieved a response to therapy (Hb increase ⩾2 g dl^−1^) compared with 27% of patients with an O/P ratio ⩾0.9 (RR 1.942; 95% CI: 1.031; 3.665; *P*<0.001). Results were similar when an alternative definition of haematopoietic response (⩾2 g dl^−1^ or reaching 12 g dl^−1^) was applied (RR 1.934; 95% CI: 1.131; 3.307; *P*<0.001).

The median Hb level before transfusion was 7.64 g dl^−1^ in the epoetin *β* group and 7.80 g dl^−1^ in the control group. Significantly fewer patients in the epoetin *β* group than the control group required transfusions of whole blood or erythrocyte concentrate during the last 8 weeks of the study (22 *vs* 43%, *P*<0.001) or over the course of the whole study (32 *vs* 52%; *P*=0.001). Overall, the mean number of units transfused patient^−1^ was reduced by 43.2% during the treatment period with epoetin *β*.

A total of 58 patients required iron supplementation during the study (epoetin *β*, *n*=30; control, *n*=28) with the majority being administered orally. A minority of patients in each group received parenteral iron (epoetin *β*, *n*=9; control, *n*=2). Patients who had iron status measured at baseline and at study end showed that those treated with epoetin *β* had a serum iron deficit of 4.5 *μ*g dl^−1^, while control patients showed a median increase of 16.8 mg dl^−1^ (*P*<0.01). No clinically relevant changes in transferrin saturation were observed for either treatment group between baseline and study end (data not shown).

#### Safety

A total of 221 patients experienced at least one adverse event during the present study (epoetin *β*, *n*=115 (86%); control, *n*=106 (82%)), leading to the withdrawal of 29 patients (epoetin *β*, *n*=19 (14%); control, *n*=10 (8%)). No relevant between-group differences were apparent in terms of the incidence or profile of specific adverse events, the majority of which were attributed to the underlying disease and/or concomitant chemotherapy (Table 5

**Table 5:** Most common adverse events^a^ (reported in ⩾5% of patients in at least one treatment group)

**Adverse event**	**Epoetin *β* (*n*=133)**	**Control (*n*=129)**
Malignancy progression	33 (25%)	42 (33%)
Anaemia	18 (14%)	33 (26%)
Leucopenia	20 (15%)	19 (15%)
Thrombocytopenia	8 (6%)	13 (10%)
Bronchitis	7 (5%)	8 (6%)
Fever	5 (4%)	10 (8%)
Nausea	6 (5%)	8 (6%)
Pain	9 (7%)	5 (4%)
Pneumonia	9 (7%)	5 (4%)
Asthenia	6 (5%)	7 (5%)
Diarrhoea	11 (8%)	2 (2%)
Infection	8 (6%)	4 (3%)
Sepsis	3 (2%)	7 (5%)
Vomiting	9 (7%)	1 (<1%)
Depression	8 (6%)	1 (<1%)
Headache	7 (5%)	2 (2%)

An adverse event was defined as any undesired, noxious or pathological change in a patient as indicated by signs, symptoms and/or laboratory changes that occurred in association with the use of a drug or placebo whether considered drug related or not.

). Few adverse events were therefore deemed to be causally related to study medication. No patient experienced local injection site reactions during s.c. administration of epoetin *β*. A serious adverse event was defined as an adverse event that was fatal or acutely life threatening, required/prolonged hospitalisation, resulted in persistent or significant disability or resulted in malignancy or congenital malformation/anomaly. Serious adverse events occurred in 42% of patients receiving epoetin *β* and 33% of patients on control therapy. The most frequent serious adverse events with epoetin *β* and control therapy were malignancy progression (10 *vs* 13%) and anaemia (8 *vs* 13%). No clinically relevant between-group differences were apparent during the study with regard to laboratory safety tests and blood pressure. In the epoetin *β* compared with the control group there was no significant difference in the mean number (±s.d.) of hospitalisations per patient (3.8±4.5 *vs* 4.1±4.9; *P*=0.52) or hospital days (11.7±13.7 *vs* 9.4±10.3 days; *P*=0.46). There was a trend towards fewer hospital admissions related to anaemia in the epoetin *β* group (0.8±2.2 *vs* 1.5±3.6; *P*=0.043).

## DISCUSSION

The anaemia of malignancy has a significant negative impact on patients' QoL. Treatment with blood transfusions is not without risk and is subject to availability of blood supplies. These factors have therefore led to the increased use of epoetin for the treatment of cancer-related anaemia. The current trial assessed the impact of epoetin *β* on QoL in patients with lymphoid or solid tumours, and showed that epoetin *β* produced improvements in QoL that were significantly correlated to the changes observed in Hb concentrations. Furthermore, epoetin *β* was well tolerated and was significantly superior to standard care in increasing Hb concentrations, irrespective of the type of tumour, or the presence and nature of chemotherapy. The findings are consistent with those reported in other clinical studies of epoetin (Case *et al*, 1993; Cascinu *et al*, 1994; Henry and Abels, 1994; Dammacco *et al*, 1998; Nowrousian, 1998; ten Bokkel Huinink *et al*, 1998; Ludwig, 1999).

Focusing on the nearly ubiquitous problems of fatigue and anaemia associated with cancer and its treatment, the FACT-F and FACT-An subscales provide additional insight about patients' QoL (Cella, 1997; Yellen *et al*, 1997). Epoetin *β* produced significant improvements in the SF-36 PCS and FACT-F subscale scores compared with blood transfusion. This finding agrees with that of a double-blind study, in which epoetin *α* produced an improvement in the SF-36 PCS score compared with placebo in anaemic cancer patients receiving nonplatinum chemotherapy (Littlewood *et al*, 2001). This improvement was not statistically significant but the study was powered to detect changes in the FACT-F questionnaire, not the SF-36 PCS.

The consistent improvement in all primary end points, and in the secondary end points, reported in this study provides further support and internal validity to the finding that epoetin *β* significantly improves QoL in anaemic cancer patients. From the perspective of external validity, the study by Österborg *et al* (2002) also revealed that epoetin *β* was associated with a statistically significant (*P*<0.05) increase in the *total* FACT-An score after 12 and 16 weeks of treatment.

In the current study, patients' self-assessment of global QoL as indicated by VAS scores showed a significant increase with epoetin *β* relative to control therapy. This is consistent with previous community-based studies of over 2000 chemotherapy recipients, showing a significant improvement in mean VAS ratings of energy, activity and overall QoL following epoetin therapy (Glaspy, 1997; Glaspy *et al*, 1997; Demetri *et al*, 1998). As in the current trial, QoL improvements in these studies also correlated significantly with Hb concentrations.

The rates of missing QoL data points at the end of the study ranged from 13 to 26%, which is highly favourable compared to other recent QoL studies in cancer patients (Gabrilove *et al*, 2001; Littlewood *et al*, 2001). Data without LOCF analysis provided larger differences in QoL scores between the two groups in favour of epoetin *β* than were shown following LOCF analysis. Thus, the QoL data from this study are robust and any conclusions drawn from the QoL data are independent of the method used to handle the missing data points.

It is possible that the nonblinded nature of this study may have influenced the increases in QoL observed, as patients would have been aware that they were receiving epoetin *β*. However, increases in the FACT-F and FACT-An scores during this study for patients with haematological malignancies are comparable with the increases observed in a recently published double-blind, placebo-controlled study examining the effects of epoetin *β* in the same patient population (Osterborg *et al*, 2002). Thus, it appears that the fact that the study was open-label did not unduly influence the reporting of QoL results.

A reliable method of predicting potential responders and nonresponders to epoetin therapy would be extremely valuable in the clinical setting, as this would enable physicians to target therapy at those patients who are most likely to benefit. A relation has been demonstrated between defective endogenous erythropoietin production and the likelihood of response to epoetin (Barosi *et al*, 1994). Furthermore, the endogenous erythropoietin O/P ratio is an important measure of endogenous erythropoietin production. In this study, in patients with lymphoid tumours, 52% of patients with a serum erythropoietin O/P ratio <0.9 achieved the expected response to therapy (Hb increase ⩾2 g dl^−1^), compared with 27% of patients with a baseline O/P ratio ⩾0.9. This is consistent with the results of previous studies in patients with multiple myeloma or non-Hodgkin's lymphoma (Cazzola *et al*, 1995; Österborg *et al*, 1996). Österborg *et al* (1996) showed that the probability of a response to epoetin was highest in patients with an O/P ratio of ⩽0.9 and Cazzola *et al* (1995) suggested that a low-serum erythropoietin level or O/P ratio should be used to identify patients very likely to respond to – and therefore good candidates for – epoetin therapy.

This study shows that it is possible to achieve a significant improvement in the functional status and QoL of cancer patients using epoetin *β*. Studies examining the cost-effectiveness of epoetin used in the cancer setting have produced mixed conclusions, but these findings are based on clinical trial results from the early-to-mid 1990s (Cremieux *et al*, 1999; Sheffield *et al*, 1997). Findings from more recently published clinical studies, including this one, suggest a need to examine the cost-effectiveness of epoetin *β* taking into consideration (1) the value of targeting patients using serologic markers at baseline and early in treatment, (2) the public concern about the safety of the blood supply and (3) the prospect that better anaemia management may improve survival.

In conclusion, the results of the current study indicate that, when compared with transfusion therapy, epoetin *β* produces a clinically significant improvement in QoL in patients with anaemia associated with malignancy. Epoetin *β* improved physical functioning and well-being as a result of diminished anaemia-related symptoms as measured by the FACT-An and FACT-F questionnaires. These improvements in QoL accompany and are mediated through improvements in Hb concentration, and can be achieved after a few weeks of epoetin *β* therapy. In addition, baseline erythropoietin serum levels and the O/P ratio might identify those patients with lymphoproliferative malignancies who are more likely to respond to epoetin *β*. However, the use of the O/P ratio to predict which patients will respond to rhEPO therapy requires further study.

## References

[bib1] Barosi G, Cazzola M, De Vincentiis A, Grossi A, Tura S (1994) Guidelines for the use of recombinant human erythropoietin. Haematologica 79: 526–533 7896210

[bib2] Beguin Y, Yerna M, Loo M, Weber M, Fillet G (1992) Erythropoiesis in multiple myeloma: defective red cell production due to inappropriate erythropoietin production. Br J Haematol 82: 648–653 1482651 10.1111/j.1365-2141.1992.tb06939.x

[bib3] Caro JJ, Salas M, Ward A, Goss G (2001) Anemia as an independent prognostic factor for survival in patients with cancer. Cancer 91: 2214–2221 11413508

[bib4] Cascinu S, Fedeli A, Del Ferro E, Luzi Fedeli S, Catalano G (1994) Recombinant human erythropoietin treatment in cisplatin-associated anemia: a randomized, double-blind trial with placebo. J Clin Oncol 12: 1058–1062 8164030 10.1200/JCO.1994.12.5.1058

[bib5] Case DC, Bukowski RM, Carey RW, Fishkin EH, Henry DH, Jacobson RJ, Jones SE, Keller AM, Kugler JW, Nichols CR, Salmon SE, Silver RT, Storniolo AM, Wampler GL, Dooley CM, Larholt KM, Nelson RA, Abels RI (1993) Recombinant human erythropoietin therapy for anemic cancer patients on combination chemotherapy. J Natl Cancer Inst 85: 801–806 8487324 10.1093/jnci/85.10.801

[bib6] Cazzola M, Beguin Y (1992) New tools for clinical evaluation of erythron function in man. Br J Haematol 80: 278–284 1581207 10.1111/j.1365-2141.1992.tb08133.x

[bib7] Cazzola M, Messinger D, Battistel V, Bron D, Cimino R, Enller-Ziegler L, Essers U, Greil R, Grossi A, Jager G, LeMevel A, Najman A, Silingardi V, Spriano M, van Hoof A, Ehmer B (1995) Recombinant human erythropoietin in the anemia associated with multiple myeloma or non-Hodgkin's lymphoma: dose finding and identification of predictors of response. Blood 86: 4446–4453 8541533

[bib8] Cazzola M, Ponchio L, Pedrotti C, Farina G, Cerani P, Lucotti C, Novella A, Rovati A, Bergamaschi G, Beguin Y (1996) Prediction of response to recombinant human erythropoietin (rHuEpo) in anemia of malignancy. Haematologica 81: 434–441 8952157

[bib9] Cella D (1997) The Functional Assessment of Cancer Therapy-Anemia (FACT-An) Scale: a new tool for the assessment of outcomes in cancer anemia and fatigue. Semin Hematol 34 (3 Suppl 2): 13–19 9253779

[bib10] Cremieux P-Y, Finkelstein SN, Berndt ER, Crawford J, Slavin MB (1999) Cost effectiveness, quality-adjusted life-years and supportive care. Recombinant human erythropoietin as a treatment of cancer-associated anaemia. Pharmacoeconomics 16: 459–472 10662393 10.2165/00019053-199916050-00004

[bib11] Curt GA, Breitbart W, Cella D, Groopman JE, Horning SJ, Itri LM, Johnson DH, Miaskowski C, Scherr SL, Portenoy RK, Vogelzang NJ (2000) Impact of cancer-related fatigue on the lives of patients: new findings from the fatigue coalition. Oncologist 5: 353–360 11040270 10.1634/theoncologist.5-5-353

[bib12] Dammacco F, Castoldi G, Rodjer S (2001) Efficacy of epoetin alfa in the treatment of anaemia of multiple myeloma. Br J Haematol 113: 172–179 11328297 10.1046/j.1365-2141.2001.02715.x

[bib13] Dammacco F, Silvestris F, Castoldi GL, Grassi B, Bernasconi C, Nadali G, Perona G, De Laurenzi A, Torelli U, Ascari E, Rossi Ferrini PL, Caligaris-Cappio F, Pileri A, Resegotti L (1998) The effectiveness and tolerability of epoetin alfa in patients with multiple myeloma refractory to chemotherapy. Int J Clin Lab Res 28: 127–134 9689556 10.1007/s005990050032

[bib14] Demetri GD, Kris M, Wade J, Degos L, Cella D (1998) Quality-of-life benefit in chemotherapy patients treated with epoetin alfa is independent of disease response or tumor type: results from a prospective community oncology study. Procrit Study Group. J Clin Oncol 16: 3412–3425 9779721 10.1200/JCO.1998.16.10.3412

[bib15] Gabrilove JL, Cleeland CS, Livingston RB, Sarokhan B, Winer E, Einhorn LH (2001) Clinical evaluation of once-weekly dosing of epoetin alfa in chemotherapy patients: improvements in hemoglobin and quality of life are similar to three-times-weekly dosing. J Clin Oncol 19: 2875–2882 11387360 10.1200/JCO.2001.19.11.2875

[bib16] Glaspy J (1997) The impact of epoetin alfa on quality of life during cancer chemotherapy: a fresh look at an old problem. Semin Hematol 34: 20–26 9253780

[bib17] Glaspy J, Bukowski R, Steinberg D, Taylor C, Tchekmedyian S, Vadhan-Raj S (1997) Impact of therapy with epoetin alfa on clinical outcomes in patients with nonmyeloid malignancies during cancer chemotherapy in community oncology practice. Procrit Study Group. J Clin Oncol 15: 1218–1234 9060566 10.1200/JCO.1997.15.3.1218

[bib18] Grogan M, Thomas GM, Melamed I, Wong FL, Pearcey RG, Jospeh PK, Portelance L, Crook J, Jones KD (1999) The importance of hemoglobin levels during radiotherapy for carcinoma of the cervix. Cancer 86: 1528–1536 10526282 10.1002/(sici)1097-0142(19991015)86:8<1528::aid-cncr20>3.0.co;2-e

[bib19] Groopman JE, Itri LM (1999) Chemotherapy-induced anemia in adults: incidence and treatment. J Natl Cancer Inst 91: 1616–1634 10511589 10.1093/jnci/91.19.1616

[bib20] Henry DH, Abels RI (1994) Recombinant human erythropoietin in the treatment of cancer and chemotherapy-induced anemia: results of double-blind and open-label follow-up studies. Semin Oncol 21: 21–28 8202722

[bib21] Kaye SB, Lewis CR, Paul J, Duncan ID, Gordon HK, Kitchener HC, Cruicjshank DJ, Atkinson RJ, Soukop M, Rankin EM et al. (1992) Randomised study of two doses of cisplatin with cyclophosphamide in epithelial ovarian cancer. Lancet 340: 329–333 1353804 10.1016/0140-6736(92)91404-v

[bib22] Littlewood TJ, Bajetta E, Nortier JWR, Vercammen E, Rapoport B for the Epoetin Alfa Study Group (2001) Effects of epoetin alfa on hematologic parameters and quality of life in cancer patients receiving nonplatinum chemotherapy: results of a randomized, double-blind, placebo-controlled trial. J Clin Oncol 19: 2865–2874 11387359 10.1200/JCO.2001.19.11.2865

[bib23] Ludwig H (1999) Epoetin in cancer-related anaemia. Nephrol Dial Transplant 14: 85–92 10334673 10.1093/ndt/14.suppl_2.85

[bib24] Manegold C (1998) The causes and prognostic significance of low hemoglobin levels in tumour patients. Strahlenther Onkol 174 (Suppl IV): 17–19 9879342

[bib25] Moullet I, Salles G, Ketterer N, Dumontet C, Bouafia F, Neidhart-Berard E-M, Thieblemont C, Felman P, Coiffier B (1998) Frequency and significance of anemia in non-Hodgkin's lymphoma patients. Ann Oncol 9: 1109–1115 9834824 10.1023/a:1008498705032

[bib26] Nowrousian MR (1998) Recombinant human erythropoietin in the treatment of cancer-related or chemotherapy-induced anaemia in patients with solid tumours. Med Oncol 15 (Suppl 1): S19–S28 9785333

[bib27] Nunnaly JC (1978) Psychometric Theory, 2nd edn. New York, London: McGraw-Hill

[bib28] Österborg A, Boogaerts MA, Cimino R, Essers V, Holowiecki J, Juliusson G, Jager G, Najman A, Peest D for the European Study Group of erythropoietin (Epoetin Beta) treatment in multiple myeloma and non-Hodgkin's lymphoma (1996) Recombinant human erythropoietin in transfusion-dependent anemic patients with multiple myeloma and non-Hodgkin's lymphoma – a randomized multicentre study. Blood 87: 2675–2682 8639883

[bib29] Österborg A, Brandberg Y, Molostova V, Iosova G, Abdulkadyrov K, Hedenus M, Messinger D for the Epoetin Beta Hematology Study Group (2002) Randomized, double-blind, placebo-controlled trial of recombinant human erythropoietin (epoetin beta) in hematological malignancies. J Clin Oncol 20: 2486–2494 12011126 10.1200/JCO.2002.08.131

[bib30] Rose EH, Abels RI, Nelson RA, McCullough DM, Lessin L (1995) The use of r-HuEpo in the treatment of anaemia related to myelodysplasia (MDS). Br J Haematol 89: 831–837 7772519 10.1111/j.1365-2141.1995.tb08421.x

[bib31] Sheffield R, Sullivan SD, Saltiel E, Nishimura L (1997) Cost comparison of recombinant human erythropoietin and blood transfusion in cancer chemotherapy-induced anemia. Ann Pharmacother 31: 15–22 8997459 10.1177/106002809703100101

[bib32] Skillings JR, Sridhar FG, Wong C, Paddock L (1993) The frequency of red cell transfusion for anemia in patients receiving chemotherapy. A retrospective cohort study. Am J Clin Oncol 16: 22–25 8424398 10.1097/00000421-199302000-00006

[bib33] ten Bokkel Huinink WW, de Swart CA, van Toorn DW, Morack G, Breed WP, Hillen HF, van der Hoeven JJM, Reed NS, Fairlamb DJ, Chan SYT, Godfrey KA, Kristensen GB, van Tinteren H, Ehmer B (1998) Controlled multicentre study of the influence of subcutaneous recombinant human erythropoietin on anaemia and transfusion dependency in patients with ovarian carcinoma treated with platinum-based chemotherapy. Med Oncol 15: 174–182 9819794 10.1007/BF02821936

[bib34] Ware JE, Kosinski M, Bayliss MS, McHorney CA, Rogers WH, Raczek A (1995) Comparison of methods for the scoring and statistical analysis of SF–36 health profile and summary measures: summary of results from the Medical Outcomes Study. Med Care 33: AS264–AS279 7723455

[bib35] Ware JE, Kosinski M, Keller SD (1994) Physical and Mental Health Summary Scales: a User's Manual. Boston MA: Health Assessment Lab

[bib36] Yellen SB, Cella DF (1995) Someone to live for: social well-being, parenthood status, and decision-making in oncology. J Clin Oncol 13: 1255–1264 7738630 10.1200/JCO.1995.13.5.1255

[bib37] Yellen SB, Cella DF, Webster K, Blendowski C, Kaplan E (1997) Measuring fatigue and other anemia-related symptoms with the functional assessment of cancer therapy (FACT) measurement system. J Pain Symptom Manage 13: 63–74 9095563 10.1016/s0885-3924(96)00274-6

